# Characterization of Lactic Acid Bacteria from Fermented Fish (*pla-paeng-daeng*) and Their Cholesterol-lowering and Immunomodulatory Effects

**DOI:** 10.1264/jsme2.ME22044

**Published:** 2023-02-09

**Authors:** Engkarat Kingkaew, Hiroshi Konno, Yoshihito Hosaka, Wongsakorn Phongsopitanun, Somboon Tanasupawat

**Affiliations:** 1 Department of Biochemistry and Microbiology, Faculty of Pharmaceutical Sciences, Chulalongkorn University, Bangkok 10330, Thailand; 2 Akita Konno Co., Ltd., 248 Aza Kariwano, Daisen-shi, Akita 019–2112, Japan

**Keywords:** lactic acid bacteria (LAB), Thai fermented fish, cholesterol-lowering effects, immunomodulation, probiotics

## Abstract

The cholesterol-lowering and immunomodulatory effects and probiotic properties of 25 lactic acid bacteria (LAB) isolated from fermented fish (*pla-paeng-daeng*) in Thailand were examined in the present study. Based on their phenotypic and genetic characteristics, LAB were identified as *Lactiplantibacillus pentosus* (Group I, 6 isolates), *Lactiplantibacillus argentoratensis* (Group II, 1 isolate), *Limosilactobacillus fermentum* (Group III, 2 isolates), *Companilactobacillus pabuli* (Group IV, 4 isolates), *Companilactobacillus farciminis* (Group V, 5 isolates), *Companilactobacillus futsaii* (Group VI, 6 isolates), and *Enterococcus lactis* (Group VII, 1 isolate). *Lactiplantibacillus pentosus* PD3-1 and PD9-2 and *Enterococcus lactis* PD3-2 exhibited bile salt hydrolase (BSH) activities. The percentage of cholesterol assimilated by all isolates ranged between 21.40 and 54.07%. Bile salt hydrolase-producing isolates tolerated acidic and bile conditions and possessed adhesion properties. They also exerted immunomodulatory effects that affected the production of interleukin-12 (IL-12), interferon-γ (IFN-γ), human β-defensin-2 (hBD-2), and nitric oxide (NO). These isolates meet standard probiotic requirements and exert beneficial effects.

*Pla-paeng-daeng* (fermented fish) is a typical fermented food of Southern Thailand. It is a reddish, semi-solid, whole fish or pieces of fish (*Nemotolosa nasus*, *Ta-pian-nam-khem*) with a sour (pH 3.5–4.0) and salty taste (2.3–4.0% NaCl) that is fermented for 4–5 days. *Saccharomyces cerevisiae*, *Staphylococcus* sp., *Bacillus* sp., *Vibrio* sp., and *Tetragenococcus halophilus* strains have been identified in‍ ‍fermented fish products (Phithakpol *et al.*, 1995; Tanasupawat and Komagata, 1995). Lactic acid bacteria (LAB) contribute to the flavor, texture, and odor of food and also enhance food preservation. They are used as probiotics in several Asian fermented foods (Ngasotter *et al.*, 2020). Some LAB are generally regarded as safe (GRAS) microorganisms (Nasrollahzadeh *et al.*, 2022). Previous studies suggested the beneficial effects of probiotics as part of a healthy diet in addition to their health-promoting effects, such as reductions in cholesterol and immunomodulation (Ohashi and Ushida, 2009; Angmo *et al.*, 2016).

The administration of LAB has been shown to reduce the risk of coronary heart disease (CAD) (De Vries *et al.*, 2006). Furthermore, a slight (1%) reduction in serum cholesterol decreased the risk of CAD by 2 to 3% (Albano *et al.*, 2018). The immunomodulatory effects of LAB have recently been attracting increasing attention. Probiotic LAB regulate immunity, exert immune-boosting effects, and are used to treat unique disorders, such as immunodeficiency and autoimmune diseases. Interferon-γ (IFN-γ) enhances a host’s defenses against intracellular infections. Interleukin-12 (IL-12) is a pro-inflammatory cytokine that plays a role in preventing infection and cancer and induces the production of IFN-γ (Thamacharoensuk *et al.*, 2017). Previous studies demonstrated that *Lactobacillus* and other LAB modulated the synthesis of IL-12 and IFN-γ (Chen *et al.*, 2013; Thamacharoensuk *et al.*, 2017; Moon *et al.*, 2019; Nakai *et al.*, 2019). Defensins are human antimicrobial peptides, which play important roles in host defenses. Human β-defensin-2 (hBD-2) is activated by infection or inflammation. The expression of BD is up-regulated by several LAB strains, which may prevent infections (Kobatake and Kabuki, 2019). Nitric oxide (NO) is an endogenously synthesized molecule that plays a vital role in defenses against infection and immunomodulation (Wang *et al.*, 2009). LAB-induced NO production has been extensively examined (Kmonickova *et al.*, 2012; Surayot *et al.*, 2014).

Thai traditional fermented food is an excellent source of potentially novel probiotic isolates. There is currently no information on the cholesterol-lowering and immunomodulatory effects of LAB in *pla-paeng-daeng*. Therefore, we herein attempted to characterize LAB from Thai fermented fish (*pla-paeng-daeng*) based on phenotypic and genotypic characteristics and examined their bile salt hydrolase (BSH) activities, cholesterol assimilation capacities, immunomodulatory effects, and probiotic properties.

## Materials and Methods

### Sources and isolation

Twelve samples of fermented fish (*pla-paeng-daeng*) were collected from various local markets in the southern part of Thailand (Table 1). Twenty-five grams or 25‍ ‍mL of each sample was enriched in 225‍ ‍mL of MRS broth (de Man, Rogosa and Sharpe; Difco) (De Man *et al.*, 1960) and incubated at 30°C for 72 h. One loopful of the culture broth was then streaked over MRS agar supplemented with 0.3% (w/v) CaCO_3_ and incubated under the same conditions. Colonies surrounded by a clear zone were selected for purification. Pure cultures were stored at –20°C in 40% (v/v) glycerol and lyophilized with 10% (w/v) skim milk. The number of viable LAB in samples was assessed by counting with colony-forming units on MRS agar plates as described above.

### Identification methods

#### Phenotypic characterization

After incubation on MRS agar plates at 30°C for 48‍ ‍h, colony appearance, cell shape, cell organization, catalase activity, and Gram staining were examined. The following physiological and biochemical characteristics were assessed according to the methods described by Tanasupawat *et al.* (1998): growth in 2, 4, 6, and 8% (w/v) NaCl, growth at temperatures of 15, 30, and 45°C, growth at pH 3.0, 6.0, and 9.0, nitrate reduction, gas production, aesculin hydrolysis, arginine hydrolysis, and acid production from carbohydrates. A hierarchical cluster ana­lysis to group isolates using SPSS version 22.0 was performed based on phenotypic characteristics.

#### Genotypic characterization

The 16S rRNA gene sequences of isolates were amplified by PCR as previously described by Phuengjayaem *et al.* (2017). PCR products were sequenced using a DNA sequencer (Macrogen) with universal primers, as reported by Lane (1991). Sequence similarity values between the isolates and their related reference isolates were calculated using the EzBiocloud tool (Yoon *et al.*, 2017). MEGA 7 constructed a phylogenetic tree using the neighbor-joining (NJ) approach (Saitou and Nei, 1987; Kumar *et al.*, 2016). A bootstrap ana­lysis with 1,000 replicates was used to assess the confidence values for each branch in the phylogenetic tree (Felsenstein, 1985). The sequences identified were deposited in the DNA Data Bank of Japan (DDBJ, Mishima, Japan) and accession numbers in the DDBJ database are shown in Table 2.

### BSH activity

BSH activity was assessed with slight modifications to the method described by Shehata *et al.* (2016). Twenty microliters of the overnight culture broth was spotted on MRS agar supplemented with 0.037% (w/v) calcium chloride (CaCl_2_) and 0.5% (w/v) taurodeoxycholic acid (TDCA) (sodium salt hydrate). Plates were incubated anaerobically at 37°C for 72 h. BSH activity was indicated by the formation of halos around colonies or white opaque colonies. Non-modified MRS served as the control. BSH-producing LAB were selected to assess probiotic characteristics.

### Cholesterol assimilation

The assimilation of cholesterol by LAB was examined in MRS broth supplemented with cholesterol-polyethylene glycol (PEG) 600 (Sigma) at a final concentration of 100‍ ‍μg mL^–1^. Each inoculum (1% [v/v]) was inoculated into MRS-cholesterol-PEG 600 and incubated anaerobically at 37°C for 24 h. Cholesterol in MRS broth was extracted using the technique described by Tomaro-Duchesneau *et al.* (2014). The residual cholesterol content was measured using the modified method of Rudel and Morris (1973). The following cholesterol concentrations were used to construct a standard absorbance curve: 0.000, 3.1250, 6.250, 12.50, 25.00, 50.00, 75.00, 100.0, and 125.0‍ ‍μg mL^–1^ in MRS. Cholesterol concentrations were compared to the standard curve constructed using the cholesterol stock solution. All experiments were performed in triplicate. The abilities of all LAB to assimilate cholesterol in MRS were shown as the percentage of cholesterol assimilated in each incubation as follows:

Cholesterol assimilated(µg mL–1)

=(Cholesterol[µg mL–1])0 h–(Cholesterol[µg mL–1])24 h

% Cholesterol assimilated

=(Cholesterol assimilated[μg mL-1]Cholesterol[μg mL-1]0 h)×100

### Evaluation of probiotic properties

#### Preparation of LAB cell suspensions

LAB cell suspensions were prepared as described by Pithva *et al.* (2014) to examine probiotic characteristics. The selected isolates were propagated twice in MRS broth at 30°C for 24 h. LAB cells were then collected by centrifugation at 9,000×*g* at 4°C for 10‍ ‍min, washed twice with phosphate buffer (0.1 M, pH 7.2, containing 0.85% [w/v] NaCl), and resuspended in phosphate buffer (0.1 M, pH 7) to obtain a cell suspension with OD_600_=1 and 10^9^‍ ‍CFU‍ ‍mL^–1^.

#### Acid and bile tolerance

The selected LAB isolates were subjected to the acid tolerance test using a modified method of Thamacharoensuk *et al.* (2017). Briefly, a LAB cell suspension was inoculated into MRS broth pH 2 and 3 or supplemented with 0.3 and 0.8% (w/v) bile salt and then incubated anaerobically at 37°C for 3 h. The number of viable LAB was then quantified using a serial 10-fold dilution and the spot plate technique, as described by Whitmire and Merrell (2012). Viable bacteria were expressed as logarithms of colony-forming units per milliliter (logCFU mL^–1^).

#### Adhesion assay

The human intestinal epithelial cell line Caco-2 was used to investigate the adhesion properties of selected LAB isolates, with slight modifications to the procedure described by Han *et al.* (2017). Caco-2 cells were routinely grown at 37°C in Dulbecco’s Modified Eagle’s Medium (DMEM) supplemented with 10% (v/v) fetal bovine serum (FBS) and 1% (v/v) penicillin-streptomycin (PS) under humidified conditions of 95% air and 5% CO_2_. Caco-2 cells were seeded on 24-well tissue culture plates at a concentration of 5×10^5^‍ ‍cells‍ ‍mL^–1^ for the adhesion test. Tissue plates were incubated at 37°C in a 5% CO_2_ incubator until Caco-2 cells developed a confluent monolayer of differentiated cells. Caco-2 cells were then washed twice in PBS. The LAB cell suspension was obtained by centrifugation at 12,100×*g* at 4°C for 10‍ ‍min and re-suspended in DMEM without antibiotics. Each LAB cell suspension was seeded on wells and incubated at 37°C for 90‍ ‍min in a 5% CO_2_ incubator. Caco-2 cells were washed three times with PBS after the incubation to remove unbound LAB cells. Cells were then lysed with 0.05% Triton X-100 solution. The number of adhering bacteria was counted using the spot plate method on MRS agar and then incubated at 37°C for 48 h. *Lacticaseibacillus rhamnosus* GG was used as the control. The adherent abilities of the selected LAB were calculated according to the following equation;

$$\text{Percentage of adhering cells (\%)}=\frac{N_{t}}{N_{0}}\times 100$$

where N_t_=the number of LAB cells adhering to Caco-2 cells

N_0_=the total number of inoculated LAB cells

### Immunomodulatory effects

The immunomodulatory effects of the selected LAB were examined according to the method of Hosaka *et al.* (2021).

### Preparation of sterilized LAB powder

Selected isolates were inoculated into MRS broth and incubated at 30°C with shaking at 120‍ ‍rpm for 24 h. After sterilizing the culture medium at 100°C for 20‍ ‍min, the selected isolates were collected by centrifugation at 1,000‍ ‍rpm for 10‍ ‍min. The selected LAB powders were washed with sterile distilled water and then lyophilized to obtain sterilized LAB powders. LAB powders were suspended in PBS at a concentration of 200‍ ‍μg mL^–1^ to prepare test samples.

### Cell culture and cell differentiation

RAW264.7 cells were cultured in DMEM supplemented with 5% FBS and 0.25% PS at 37°C in a 5% CO_2_ incubator. Professor Shinichi Yokota of Sapporo Medical University School of Medicine donated Caco-2 cells. Caco-2 cells were grown in DMEM supplemented with 5% FBS and 0.25% PS at 37°C in a 5% CO_2_ incubator. THP-1 cells were grown in RPMI 1640 media supplemented with 10% FBS and 0.20% PS at 37°C in a 5% CO_2_ incubator.

Caco-2 cells (1.5×10^5^ cells) were cultivated for 72‍ ‍h in cell culture inserts (24-well hanging inserts 0.4 m; Falcon). After 72 h, the solution containing 5‍ ‍mM sodium butyrate was replaced, and cells were cultured for 96‍ ‍h to promote differentiation. Differentiated cells were evaluated by transepithelial electrical resistance (TEER) using Millicell-ERS (Merk), and differentiated cells (TEER values >400‍ ‍Ω cm^2^) were used. THP-1 cells were seeded on a multi-well plate (24-well; Falcon) and incubated for 72‍ ‍h in media supplemented with 100‍ ‍ng mL^–1^ cholecalciferol (Vitamin D_3_) and 10 nM phorbol 12-myristate13-acetate (PMA) to differentiate into macrophage-like cells. Caco-2 and THP-1 cells were then co-cultured in transwells.

### Measurement of NO production

The production of NO was measured as described by Yang *et al.* (2018). RAW264.7 cells were suspended in DMEM medium (5% FBS+0.2% PS) at a concentration of 3×10^5^‍ ‍cells‍ ‍mL^–1^, seeded on a 24-well plate, and incubated at 37°C for 24‍ ‍h in a 5% CO_2_ incubator. Samples were examined at a final concentration of 20‍ ‍μg mL^–1^ to stimulate cells. PBS was used as a negative control and LPS (10‍ ‍μg mL^–1^) (Fujifilm Wako) as a positive control. After a 24-h stimulation, the supernatant was collected, centrifuged at 12,100×*g* for 20‍ ‍min, and subjected to the Griess reaction as reported by Baek *et al.* (2015). One hundred microliters each of Griess reagent, medium supernatant sample, and 1.56 to 100‍ ‍μM of sodium nitrite standard solution were added to 96-well microplates and incubated at room temperature for 20‍ ‍min. Absorbance at 550‍ ‍nm was measured by a microplate reader. The quantity of nitrite in the medium supernatant was enumerated using a calibration curve generated from sodium nitrite standard solution.

### Intestinal immunity model

An *in vitro* intestinal immune model was generated by a co-culture of cell culture inserts (apical side) and multi-well plates (basal side). Samples were suspended in RPMI 1640 medium on the apical side (final concentration 20‍ ‍μg mL^–1^), and cells were stimulated at 37°C for 2 days in a 5% CO_2_ incubator. The basal side of the medium was then collected, and after centrifugation at 12,100×*g* for 20‍ ‍min, the supernatant was harvested to eliminate foreign compounds. Regarding IL-12 and IFN-γ, proteins were precipitated by adding a 25% volume of 100% trichloroacetic acid (TCA) to the culture medium supernatant. Precipitates were cleaned with acetone to eliminate TCA and then dissolved in 1× sample buffer for protein enrichment after a heat treatment at 100°C for 2‍ ‍min. SDS-PAGE were used to separate proteins and was performed according to Laemmli (1970). Target proteins were detected by Western blotting as previously described by Towbin *et al.* (1979). Calibration curves were prepared with known concentrations of the IL-12 standard (Gibco) and IFN-γ standard (Gibco) to calculate the production of IL-12 and IFN-γ. Production levels were corrected by measuring β-actin as an endogenous control. Regarding hBD-2, an unenriched medium supernatant was analyzed by a dot blot, and the amount of hBD-2 produced was corrected from the amount of total protein by CBB staining. Values were evaluated relative to the non-stimulated test section with PBS.

### Statistical ana­lysis

All experiments were performed in triplicate, and the results obtained are shown as the mean±standard deviation (SD). Results on acid and bile tolerance as well as adhesion ability were examined by ANOVA using SPSS 22.0 software. Duncan’s Multiple Range Test (DMRT) was used for comparisons of mean values at a significance level of *P*<0.05. Immunomodulatory effects were assessed by Welch’s *t*-test at a significance level of *P*<0.05.

## Results and Discussion

### Isolation and identification of isolates

**Fig. 2.**
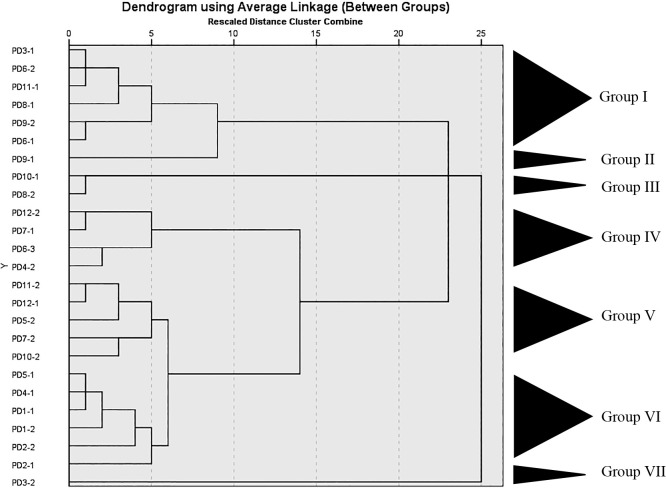
Dendrogram of hierarchical cluster-based phenotypic characteristics.

**Fig. 3.**
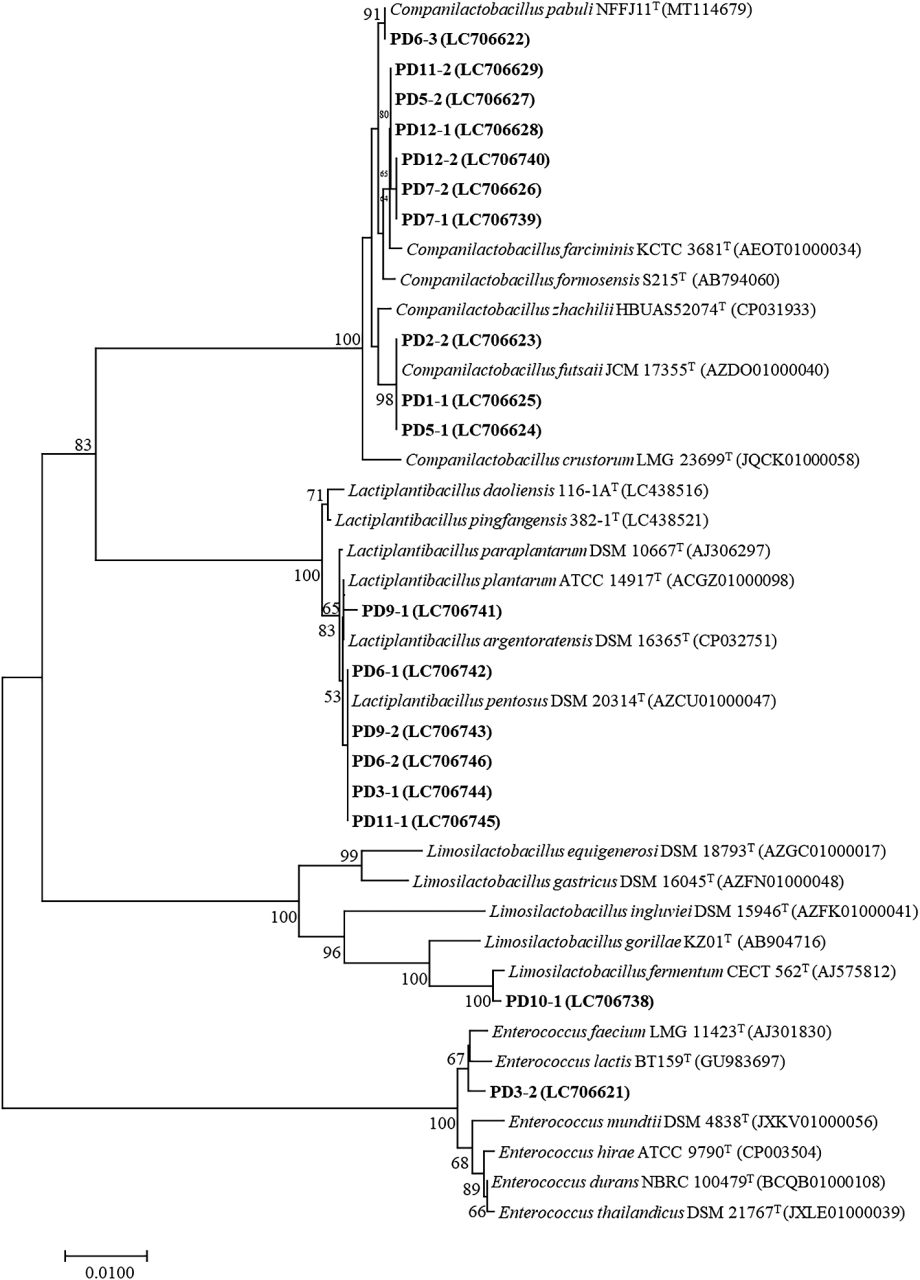
Neighbor-joining tree based on the 16S rRNA gene of representative isolates from each group.

The total number of LAB detected in 6 samples collected from Nakhon Si Thammarat, 3 from Songkhla, and 2 from Satul Province ranged between 1.6×10^4^ and 5.7×10^8^, 4.3×10^5^ and 1.3×10^8^, and 1.4×10^7^ and 2.2×10^9^ CFU g^–1^, respectively. Twenty-five LAB isolates were isolated from Thai fermented fish (*pla-paeng-daeng*) samples based on differences in colony appearance and cell form (Table 1). All isolates were Gram-positive, catalase-negative, and facultatively anaerobic bacteria. They were members of the genera *Lactiplantibacillus*, *Limosilactobacillus*, *Companilactobacillus*, and *Enterococcus*, were divided into 7 groups according to the results of a hierarchical cluster ana­lysis of their phenotypic characteristics, and 16S rRNA gene sequence similarities among the representative isolates were examined (Fig. 2, 3, and Table 2).

Group I included six rod-shaped isolates (PD3-1, PD6-2, PD11-1, PD8-1, PD9-2, and PD6-1). They did not produce gas from glucose. They grew at pH 3 and 9, in 6 and 8% NaCl, and at 15°C, but not at 45°C. They reduced nitrate, but did not hydrolyze arginine. The isolates contained *meso*-DAP in their cell walls. They produced DL-lactic acid. Acid production from L-arabinose, D-galactose, D-melibiose, D-raffinose, and D-ribose varied. The representative isolates in this group showed 99.71 to 100% 16S rRNA gene sequence similarity (Table 2) to *Lactiplantibacillus pentosus* DSM 20314^T^ (Fig. 2). Therefore, they were closely related to *Lb. pentosus* and their differential phenotypic characteristics are shown in Table 3. *Lb. pentosus* strains have mostly been found in fermented fish products (Rodpai *et al.*, 2021; Syafitri *et al.*, 2022).

Group II included one rod-shaped isolate (PD9-1). It did not produce gas from glucose. It grew at pH 3 and 9, 15 and 45°C, and in 6 and 8% NaCl. It contained *meso*-DAP in its cell walls. It produced DL-lactic acid. It did not produce acid from aesculin. It did not hydrolyze arginine. It did not reduce nitrate. The representative isolate in this group showed 99.85% 16S rRNA gene sequence similarity (Table 2) to *Lactiplantibacillus argentoratensis* DSM 16365^T^ (Fig.‍ ‍2). Therefore, it was identified as *Lactiplantibacillus argentoratensis* and its phenotypic characteristics are shown in Table 3.

Group III included two rod-shaped isolates (PD10-1 and PD8-2). They produced gas from glucose. They grew at pH 3 and in 8% NaCl, but did not grow at pH 9. These isolates did not contain *meso*-DAP in their cell walls. They did not reduce nitrate. They produced DL-lactic acid. They hydrolyzed arginine. Neither of the isolates produced acid from D-cellobiose, D-galactose, lactose, D-mannose, D-mannitol, D-raffinose, L-rhamnose, salicin, D-sorbitol, D-trehalose, or aesculin. The representative isolate in this group showed 99.49% 16S rRNA gene sequence similarity (Table 2) to *Limosilactobacillus fermentum* CECT 562^T^ (Fig. 2). Therefore, they were identified as *Limosilactobacillus fermentum* and their differential phenotypic characteristics are shown in Table 3.

Group IV included four rod-shaped isolates (PD12-2, PD7-1, PD6-3, and PD4-2). They did not produce gas from glucose. They grew at pH 3, at temperatures of 9, 15 and 45°C, and in 6 and 8% NaCl. They did not reduce nitrate. The isolates did not have *meso*-DAP in their cell walls. They produced DL-lactic acid. They did not produce acid from D-cellobiose, lactose, D-maltose, D-mannitol, D-melibiose, L-rhamnose, D-ribose, or D-trehalose. Variable acid production from D-xylose and aesculin was noted. They hydrolyzed arginine. The representative isolates in this group showed 99.63 to 99.71% 16S rRNA gene sequence similarity (Table 2) to *Companilactobacillus pabuli* NFFJ11^T^ (Fig. 2). Therefore, they were identified as *Companilactobacillus pabuli* and their differential phenotypic characteristics are presented in Table 3.

Group V included five rod-shaped isolates (PD11-2, PD12-1, PD5-2, PD7-2, and PD10-2). They did not produce gas from glucose. They grew at pH 3 and 9, at temperatures of 15 and 45°C, and in 6% and 8% NaCl. They hydrolyzed arginine. They did not reduce nitrate. The isolates did not have *meso*-DAP in their cell walls. They produced L-lactic acid. They did not produce acid from L-arabinose, D-cellobiose, lactose, D-mannitol, D-melibiose, D-raffinose, L-rhamnose, D-ribose, D-sorbitol, D-trehalose, or D-xylose. Acid production from D-maltose, salicin, sucrose, and aesculin varied. The representative isolates in this group showed 99.77% to 99.85% 16S rRNA gene sequence similarity (Table 2) to *Companilactobacillus farciminis* KCTC 3681^T^ (Fig. 2). Therefore, they were identified as‍ ‍*Companilactobacillus farciminis* and their differential phenotypic characteristics are shown in Table 3.

Group VI included six rod-shaped isolates (PD5-1, PD4-1, PD1-1, PD1-2, PD2-2, and PD2-1). They did not produce gas from glucose. They grew at pH 3 and 9, at temperatures of 15 and 45°C, and in 6 and 8% NaCl. They variably hydrolyzed arginine. The isolates did not have *meso*-DAP in their cell walls. They produced L-lactic acid. They did not reduce nitrate. None of the isolates produced acid from D-cellobiose, D-maltose, D-mannitol, D-melibiose, D-raffinose, L-rhamnose, D-ribose, D-sorbitol, D-trehalose, or D-xylose. Acid production from L-arabinose, D-galactose, lactose, and aesculin varied. Representative isolates showed 100% 16S rRNA gene sequence similarity (Table 2) to *Companilactobacillus futsaii* JCM 17355^T^ (Fig. 2). Therefore, they were identified as *Companilactobacillus futsaii* and their differential phenotypic characteristics are shown in Table 3.

Group VII included one coccal isolate (PD3-2). It did not produce gas from glucose. It grew at pH 3 and 9, at temperatures of 15 and 45°C, and in 6 and 8% NaCl. It hydrolyzed arginine. The isolate did not have *meso*-DAP in its cell walls. Nitrate reduction was not observed. It produced L-lactic acid. The isolate did not produce acid from D-raffinose, L-rhamnose, D-sorbitol, sucrose, or D-xylose. The representative isolate PD3-2 showed 99.54% 16S rRNA gene sequence similarity (Table 2) to *Enterococcus lactis* BT159^T^ (Fig. 2). Therefore, it was identified as *En. Lactis* and its differential phenotypic characteristics are shown in Table 3.

Based on the results of phenotypic characteristics, the hierarchical cluster ana­lysis, and 16S rRNA gene sequences, LAB belonged to the genus *Companilactobacillus*, followed by *Lactiplantibacillus*, *Limosilactobacillus*, and *Enterococcus*; they were identified as *Companilactobacillus futsaii*, *Companilactobacillus farciminis*, *Companilactobacillus pabuli*, *Lb. pentosus*, *Lactiplantibacillus argentoratensis*, *Limosilactobacillus fermentum*, and *En. lactis*. Microbial distribution is influenced by several factors, such as the type of fish, other ingredients, the source of fishes and ingredients, the fermentation time, and processing conditions. Traditional fish fermentation depends on spontaneous fermentation started by naturally occurring microorganisms, primarily LAB, which are present in the ingredients, in the processing facilities, and in the surrounding environment as natural starters (Valyasevi and Rolle, 2002; Visessanguan *et al.*, 2004; Ngasotter *et al.*, 2020). Fermented fish were collected from different provinces in Thailand, including the central part, Bangkok and Nonthaburi; the northeastern part, Ubon Ratchathani, Surin, and Chaiyaphum; and the northern part, Nakhonsawan, Chiangmai, and Chiangrai. Isolates of *Lb. farciminis* was obtained from *pla-ra*, *pla-chom*, *kung-chom*, and *hoi-dong*, which contained high concentrations of NaCl (>8%), isolates of *Lactobacillus* sp. were only found in *pla-ra* and *pla-chom*, and isolates of *Lb. pentosus* and *Lb. plantarum* were detected in *pla-chom* and *kung-chom*, which contained less NaCl (<8%) (Tanasupawat *et al.*, 1998). *Lb. brevis*, *Lb. casei*, *Lb. curvatus*, *Lb. farciminis*, *Lb. pentosus*, *Lb. plantarum*, *Lc. lactis*, and *Leuconostoc* spp. were detected in *som-fak*, *pla-ra*, and *pla-chom* collected in the Lopburi and Bangkok provinces (Paludan-Müller *et al.*, 1999; Ngasotter *et al.*, 2020). LAB primarily contribute to sensorial properties via acidification (Huang *et al.*, 2021). In addition, LAB metabolism prevents the growth of pathogenic and spoilage microflora and contributes to color stabilization and texture improvements (Fadda *et al.*, 2010). *Lactobacillus* isolates are often found in fermented fish products (Syafitri *et al.*, 2022). *Enterococcus* spp. may also be isolated and contribute to the development of organoleptic profiles (Comi *et al.*, 2005).

Therefore, several isolates of *Lb. pentosus* (Group I) and *Companilactobacillus farciminis* (=*Lb. farciminis*, Group V) were isolated from the Nakhon Si Thammarat, Satul, and Songkhla provinces, whereas *Companilactobacillus pabuli* (Group IV) was isolated from the Nakhon Si Thammarat and Satul provinces. However, *Companilactobacillus futsaii* (Group VI) and *En. lactis* (VII) were only found in the‍ ‍Nakhon Si Thammarat province. *Lactiplantibacillus argentoratensis* (Group II) and *Limosilactobacillus fermentum* (=*Lb. fermentum*, Group III) were isolated from the Songkhla province. Since each province has specific raw materials and fermentative procedures, the distribution and viability of LAB differed, as shown in Table 1.

### BSH activity

BSH activity is associated with reductions in cholesterol and is also recognized as an additional criterion for the selection of probiotics (Miremadi *et al.*, 2014). By deconjugating bile salts, BSH activity enhances bacterial growth and colonization in the gut (Begley *et al.*, 2006). Among 3 isolates, PD3-1 and PD9-2 exhibited BSH activity by developing opaque white colonies, whereas PD3-2 created halos around colonies (Table 2). The formation of opaque white colonies or bile acid precipitates around colonies is considered to reflect BSH activity (Dashkevicz and Feighner, 1989; Jayashree *et al.*, 2014). Therefore, BSH-producing isolates were selected to examine probiotic characteristics. These BSH-positive isolates were identified as *Lb. pentosus* PD3-1 and PD9-2 (100% similarity) and *En. lactis* PD3-2 (99.54% similarity). BSH activity by probiotic LAB contributes to reductions in serum cholesterol levels and increases resistance to bile salts (Noriega *et al.*, 2006). Based on the screening, the findings are consistent with previous studies (Abushelaibi *et al.*, 2017; Liu *et al.*, 2017), which showed that the hypocholesterolemic effects of LAB reduced serum cholesterol levels in an *in vivo* model and LAB isolated from food products exhibited BSH activity. The present results demonstrated that BSH-producing isolates were also present in non-human sources.

### Cholesterol assimilation

Hypercholesterolemia is a cause of cardiovascular disease (CVD), the leading cause of mortality (Labarthe and Dunbar, 2012). Therefore, reductions in serum cholesterol levels are vital for the prevention of CVD. In the present study, the percentage of cholesterol assimilation by all isolates ranged between 21.40 and 54.07% (Table 2). The percentage of cholesterol assimilation by only one isolate was higher than 50%, namely, *Companilactobacillus pabuli* isolate PD6-3 at 54.07%.

The amount of cholesterol assimilated markedly differed among isolates. The ability to assimilate cholesterol in the present study was consistent with previous studies (Miremadi *et al.*, 2014; Tomaro-Duchesneau *et al.*, 2014; Shehata *et al.*, 2016), which reported the cholesterol assimilation ability of LAB and variations in this ability. Cholesterol assimilation and BSH activity are cholesterol removal mechanisms and desirable characteristics for probiotics (Ishimwe *et al.*, 2015). Since LAB probiotics consume cholesterol for metabolism, luminal cholesterol levels accessible for absorption are reduced (Bordoni *et al.*, 2013).

### Acid and bile tolerance

One of the essential properties of probiotic bacteria is acid and bile tolerance, which influences their capacity to exist in the acidic stomach and small intestine and, thus, their ability to accomplish their functional activity as a probiotic (Tannock, 2004; Ruiz *et al.*, 2013). The effects of acidic and bile conditions on the survival of selected LAB are shown in Table 4. Based on BSH-positive activity, all BSH-positive isolates were selected to assess acid and bile tolerance. Under acidic conditions, the selected isolates tolerated pH 2 and 3. The viability of all isolates was significantly lower than that of the MRS control (24.20–38.39% reduction). This result is consistent with previous findings (Hassanzadazar *et al.*, 2012), showing that acidic pH environments limit metabolism by and decrease the growth and viability of LAB. Resistance at pH 3 was previously established as a criterion for the acid tolerance of probiotics (Liong and Shah, 2005).

In bile conditions, all isolates survived in the presence of different percentages of bile salts with varying degrees of viability (Table 4). The viability of isolates significantly differed from that of the MRS control. The viability of isolate PD9-2 decreased in the presence of 0.3 and 0.8% bile salts (16.90 and 17.99% reductions, respectively). Conversely, the viabilities of *En. lactis* PD3-2 and *Lb. pentosus* PD3-1 increased (+4.16-12.71% reduction) with significant differences from the MRS control. This result is in accordance with previous findings (Thamacharoensuk *et al.*, 2017), which demonstrated that high pH environments and bile salts supported the viability of some LAB isolates, but also limited the viability of other LAB isolates. Therefore, LAB in the present study survived and propagated under acidic and bile conditions, and bile salts may enhance the viability of some LAB.

Due to their resistance to acidic conditions and bile salts, these isolates may survive in the stomach and intestines or even compete with other bacterial groups and colonize the gastrointestinal tract (GIT), indicating good probiotic potential.

### Adhesion

**Fig. 4.**
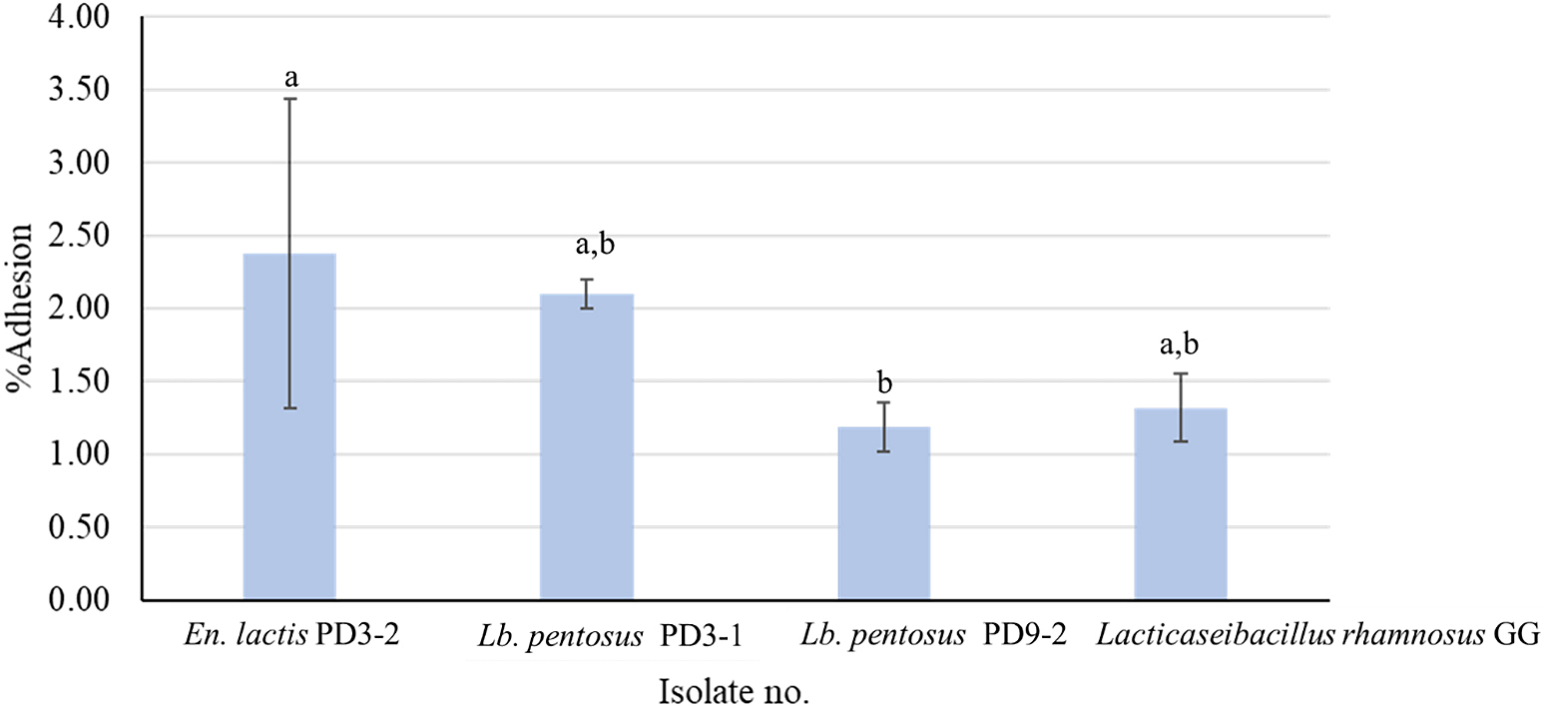
Percentage of selected isolates adhering to Caco-2 cell lines. Selected isolates were enumerated by bacterial cultures and interpreted as percent adherence relative to the control. Data represent the mean±SD. Different letters indicate a significant difference (*P*<0.05).

One of the essential properties of probiotics is their capacity to adhere to target sites for colonization in the digestive tract. Based on BSH-positive activity and acid and bile tolerance, three isolates were selected to assess *in vitro* adhesion properties: *Lb. pentosus* PD3-1 and PD9-2, and *En. lactis* PD3-2. The adhesion abilities of the selected isolates are shown in Fig. 4. The percentages of *En. lactis* PD3-2, *Lb. pentosus* PD3-1, *Lb. pentosus* PD9-2, and *Lb. rhamnosus* GG adhering to cells were 2.38±1.06, 2.10±0.10, 1.19±0.17, and 1.32±0.23%, respectively. The adhesion abilities of the selected isolates did not significantly different from that of *Lb. rhamnosus* GG. The adhesion ability of LAB in the present study was consistent with that in previous studies (Duary *et al.*, 2011; García-Cayuela *et al.*, 2014; Thamacharoensuk *et al.*, 2017), which showed that the adhesion abilities of LAB to human intestinal epithelium cells, such as Caco-2 cells, were dependent on the strain and bacterial cell-surface composition.

The present results indicated the strain-specific adhesion abilities of *Lacticaseibacillus* and *Enterococcus* isolates to Caco-2 cells, which varied within the same species (Duary *et al.*, 2011).

In summary, the selected isolates (PD3-1, PD9-2, and PD3-2) from fermented fish (*pla-paeng-daeng*) samples demonstrated a superior ability to *Lb. rhamnosus* GG (one of the most widely marketed and researched probiotic isolates [Stage *et al.*, 2020]) to adhere to Caco-2 cells *in vitro*. These isolates exhibited excellent capabilities and, thus, have potential as candidate probiotics. Further *in vivo* studies are needed to assess their health-promoting effects because they have the ability to colonize the gut.

### Immunomodulatory effects of LAB

The present results revealed that the immunomodulatory effects of the selected isolates (based on BSH-positive activity) significantly differed from those of the control (Table 5).

*En. lactis* PD3-2 was the strongest inducer of IL-12 production (57.45±7.22‍ ‍ng mL^–1^), whereas *Lb. pentosus* PD3-1 suppressed its production (7.72±2.85‍ ‍ng mL^–1^). The capacity of LAB to induce IL-12 in the present study was consistent with previous findings (Iwabuchi *et al.*, 2012; Chen *et al.*, 2013; Thamacharoensuk *et al.*, 2017), which showed that dead and viable LAB cells both modulated IL-12 levels.

Regarding IFN-γ, *En. lactis* PD3-2 was the strongest inducer of IFN-γ production (53.88±13.80‍ ‍ng mL^–1^), whereas *Lb. pentosus* PD3-1 suppressed its production (32.91±5.79‍ ‍ng mL^–1^). These results are consistent with previous findings (Ou *et al.*, 2011; Yamane *et al.*, 2018), showing that LAB cells function as immunomodulatory agents that may suppress and stimulate the production of IFN-γ.

Concerning hBD-2 production, *in vitro* results revealed that all of the selected isolates up-regulated the expression of hBD-2. These results are in accordance with previous studies (Schlee *et al.*, 2008; Kobatake and Kabuki, 2019), which reported that LAB up-regulated the expression of hBD-2 in order to prevent infection. Therefore, the present results suggest that various non-pathogenic probiotic bacteria, including LAB, promote innate immunity through the induction of defensin. Furthermore, the probiotic activation of defensins may be an appealing novel approach to strengthen innate immunity (Schlee *et al.*, 2008).

Regarding NO production, NO boosts a host’s defenses against infections and tumor cells. The results of the NO assay revealed that all of the selected isolates induced NO production at levels that significantly differed from those of the control (Table 4). *Lb. pentosus* PD9-2 induced the highest level of NO production (19.13±0.20‍ ‍μM), followed by *Lb. pentosus* PD3-1 (18.07±0.25‍ ‍μM) and *En. lactis* PD3-2 (8.30±0.09‍ ‍μM). LAB-induced NO production levels in the present study are consistent with those in previous studies (Korhonen *et al.*, 2001; Kmonickova *et al.*, 2012; Surayot *et al.*, 2014), showing that cell components of LAB stimulated various NO production levels and important factors were the type of cell component (*i.e.*, carbohydrates, lipids, and proteins) and the strain.

In the present study, non-viable cells of LAB still exerted immunomodulatory effects; therefore, the advantages of dead/dormant cells of probiotics include a reduced risk of probiotic sepsis and drug resistance as well as a longer shelf-life because storage is not necessary to maintain the viability of probiotics (Shripada *et al.*, 2020; Zendeboodi *et al.*, 2020). The present study revealed that bacterial isolates have various functional properties even if they are of the same species (Kang *et al.*, 2021a). Therefore, these isolates may stimulate immunity and protect against invading pathogens (Kato *et al.*, 1999; Kang *et al.*, 2021b).

## Conclusions

This is the first study on the distribution and characteristics of LAB in *pla-paeng-daeng*, which included *Companilactobacillus*, *Lactiplantibacillus*, *Limosilactobacillus*, and *Enterococcus* species. Two *Lb. pentosus* isolates and one *En. lactis* isolate exhibited BSH activity by forming opaque white colonies and halos around the colonies, respectively. They not only tolerated, but grew under acidic (pH 2 and 3) and bile salt (0.3 and 0.8%) conditions. They also adhered well to Caco-2 cells. Furthermore, BSH-producing isolates exerted immunostimulatory effects. *En. lactis* PD3-2 strongly induced the production of IL-12 and IFN-γ. *Lb. pentosus* PD9-2 appeared to have enhanced the secretion of hBD-2 and production of NO. The role of LAB in the prevention of hypercholesterolemia and immunomodulation is currently being investigated. Therefore, these isolates have potential as probiotics because they reduce cholesterol, exert immunomodulatory effects, show adhesion abilities, and tolerate acid and bile conditions, all of which are essential probiotic characteristics. Further clinical studies are required.

## Citation

Kingkaew, E., Konno, H., Hosaka, Y., Phongsopitanun, W., and Tanasupawat, S. (2023) Characterization of Lactic Acid Bacteria from Fermented Fish (*pla-paeng-daeng*) and Their Cholesterol-lowering and Immunomodulatory Effects. *Microbes Environ ***38**: ME22044.

https://doi.org/10.1264/jsme2.ME22044

## Figures and Tables

**Fig. 1.**
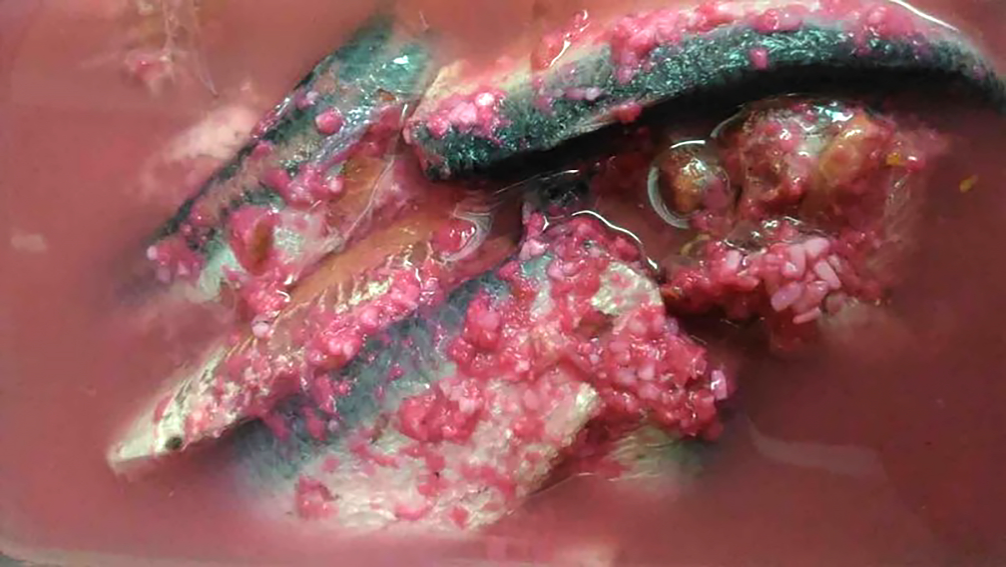
Fermented fish (*Pla-paeng-daeng*)

**Table 1.** Sample number, province, total count, and isolate number of LAB from fermented fish.

Sample no.	Province	LAB count (CFU g^–1^)	Isolate no.	Number of isolates
PD1	Nakhon Si Thammarat	1.1×10^8^	PD1-1, PD1-2	2
PD2	Nakhon Si Thammarat	5.7×10^8^	PD2-1, PD2-2	2
PD3	Nakhon Si Thammarat	1.2×10^8^	PD3-1, PD3-2	2
PD4	Nakhon Si Thammarat	2.2×10^7^	PD4-1, PD4-2	2
PD5	Nakhon Si Thammarat	1.2×10^8^	PD5-1, PD5-2	2
PD6	Nakhon Si Thammarat	2.3×10^7^	PD6-1, PD6-2, PD6-3	3
PD7	Nakhon Si Thammarat	1.6×10^4^	PD7-1, PD7-2	2
PD8	Songkhla	1.4×10^6^	PD8-1, PD8-2	2
PD9	Songkhla	1.3×10^8^	PD9-1, PD9-2	2
PD10	Songkhla	4.3×10^5^	PD10-1, PD10-2	2
PD11	Satul	2.2×10^9^	PD11-1, PD11-2	2
PD12	Satul	1.4×10^7^	PD12-1, PD12-2	2
Total				25

**Table 2.** Isolate number, nearest relatives, 16S rRNA gene sequence similarity (%), cholesterol assimilation, and BSH activity of isolates.

Isolate no.	Nearest relatives	Similarity (%)	Length (bp)	Accession no.	Cholesterol assimilation (%)	BSH activity
**Group I**						
PD3-1	*Lactiplantibacillus pentosus* DSM 20314^T^	100	1,353	LC706744	28.07±5.03	+
PD6-2	*Lactiplantibacillus pentosus* DSM 20314^T^	100	1,410	LC706746	43.40±10.39	–
PD11-1	*Lactiplantibacillus pentosus* DSM 20314^T^	99.71	1,376	LC706745	29.40±4.00	–
PD8-1		ND	ND	ND	26.07±8.08	–
PD9-2	*Lactiplantibacillus pentosus* DSM 20314^T^	100	1,354	LC706743	27.40±2.00	+
PD6-1	*Lactiplantibacillus pentosus *DSM 20314^T^	100	1,368	LC706742	45.40±5.29	–
**Group II**						
PD9-1	*Lactiplantibacillus argentoratensis* DSM 16365^T^	99.85	1,356	LC706741	47.40±8.72	–
**Group III**						
PD10-1	*Limosilactobacillus fermentum* CECT 562^T^	99.49	1,369	LC706738	48.07±4.16	–
PD8-2		ND	ND	ND	46.73±4.16	–
**Group IV**						
PD12-2	*Companilactobacillus pabuli* NFFJ11^T^	99.71	1,356	LC706740	46.07±5.03	–
PD7-1	*Companilactobacillus pabuli* NFFJ11^T^	99.71	1,370	LC706739	46.73±6.11	–
PD6-3	*Companilactobacillus pabuli* NFFJ11^T^	99.63	1,343	LC706622	54.07±13.32	–
PD4-2		ND	ND	ND	45.40±6.00	–
**Group V**						
PD11-2	*Companilactobacillus farciminis* KCTC 3681^T^	99.85	1,354	LC706629	21.40±4.00	–
PD12-1	*Companilactobacillus farciminis* KCTC 3681^T^	99.85	1,368	LC706628	27.40±3.46	–
PD5-2	*Companilactobacillus farciminis* KCTC 3681^T^	99.85	1,368	LC706627	46.07±4.16	–
PD7-2	*Companilactobacillus farciminis* KCTC 3681^T^	99.77	1,323	LC706626	32.07±3.06	–
PD10-2		ND	ND	ND	40.07±9.87	–
**Group VI**						
PD5-1	*Companilactobacillus futsaii* JCM 17355^T^	100	1,369	LC706624	32.73+4.62	–
PD4-1		ND	ND	ND	25.40±8.00	–
PD1-1	*Companilactobacillus futsaii* JCM 17355^T^	100	1,353	LC706625	45.40±2.00	–
PD1-2		ND	ND	ND	40.07±9.02	–
PD2-2	*Companilactobacillus futsaii* JCM 17355^T^	100	1,356	LC706623	34.07+5.03	–
PD2-1		ND	ND	ND	26.73±5.03	–
**Group VII**						
PD3-2	*Enterococcus lactis* BT159^T^	99.54	1,313	LC706621	32.40±9.17	+

Data on cholesterol assimilation ability are represented as the mean±SD. ND, not determined for the 16S RNA gene sequence. Bile salt hydrolase activity: +, positive reaction; –, negative reaction.

**Table 3.** Phenotypic characteristics of isolates.

**Characteristics**	I	II	III	IV	V	VI	VII
No. of isolates	6	1	2	4	5	6	1
Cell shape	Rods	Rods	Rods	Rods	Rods	Rods	Cocci in chains
Gas from glucose	–	–	+	–	–	–	–
Growth in 6% NaCl	+	+	+	+	+	+	+
Growth in 8% NaCl	+	+	+	+	+	+	+
Growth at pH 3	+	+	+	+	+	+	+
pH 9	+	+	–	+	+	+	+
Growth at 15°C	+	+	+	+	+	+	+
45°C	–	+	+	+	+	+	+
Arginine hydrolysis	–	–	+	+	+	+(–1)	–
Nitrate reduction	+	–	–	–	–	–	–
**Acid from:**							
Aesculin	+	–	–	+(–2)	–(+1)	–(+1)	+
L-Arabinose	+(–2)	+	+	+	–	–(+2)	+
D-Cellobiose	+	+	–	–	–	–	+
Fructose	+	+	+	+	+	+	+
D-Galactose	+(–2)	+	–	+	+	–(+2)	+
D-Glucose	+	+	+	+	+	+	+
Lactose	+	+	–	–	–	–(+1)	+
D-Mannose	+	+	–	+	+	+	+
D-Maltose	+	+	+	–	–(+2)	–	+
D-Mannitol	+	+	–	–	–	–	+
D-Melibiose	+(–2)	+	+	–	–	–	+
D-Raffinose	+(–3)	+	–	+	–	–	–
L-Rhamnose	+	+	–	–	–	–	–
D-Ribose	+(–1)	+	+	–	–	–	+
Salicin	+	+	–	+	+(–2)	+	+
D-Sorbitol	+	+	–	+	–	–	+
Sucrose	+	+	+	+	–(+1)	+	–
D-Trehalose	+	+	+	+(–2)	-	–	–
D-Xylose	+	+	+	+(–2)	–(+1)	–(+1)	+
*meso*-DAP	+	+	–	–	–	–	–
Isomer of lactic acid	DL	DL	DL	DL	L	L	L

+, positive reaction; –, negative reaction. Numbers in parentheses indicate the number of isolates showing the reaction.

**Table 4.** Survival of selected isolates after an incubation at various pH and % bile for 3 h.

**Isolate no.**	**Number of bacteria (logCFU mL^–1^)**						**% Reduction^b^**			
	**MRS^a^**	**pH 2**	**pH 3**	**0.3% Bile**	**0.8% Bile**		**pH 2**	**pH 3**	**0.3% Bile**	**0.8% Bile**
PD3-2	8.18±0.09	5.52±0.07*	6.15±0.07*	9.22±0.04*	9.11±0.03*		32.52	24.82	+12.71	+11.37
PD3-1	8.18±0.15	5.80±0.18*	6.02±0.21*	8.77±0.07*	8.52±0.07*		29.10	26.41	+7.21	+4.16
PD9-2	9.17±0.08	5.65±0.16*	6.95±0.31*	7.62±0.15*	7.52±0.07*		38.39	24.20	16.90	17.99

Data are expressed as the mean±SD. *P<0.05, significantly different from the negative control. ^a^ MRS was used as a negative control. ^b^ Percent reduction in the bacterial number relative to the negative control; +, indicates enhanced bacterial viability.

**Table 5.** Immunomodulatory effects of selected isolates.

**Species/isolate no.**	**IL-12 (ng mL** ^–1^ **)**	**IFN-γ (ng mL** ^–1^ **)**	**hBD-2 (relative value)**	**NO (μM)**
*En. lactis* PD3-2	57.45±7.22*	53.88±13.80*	1.96±0.10*	8.30±0.09**
*Lactiplantibacillus pentosus* PD3-1	7.72±2.85*	32.91±5.79*	2.06±0.27*	18.07±0.25**
*Lb. pentosus* PD9-2	15.38±4.93*	33.95±7.93*	2.45±0.25*	19.13±0.20**
PBS (no stimulation)	29.52±5.87	43.23±12.72	1.00±0.00	Not detected
LPS (positive control)	Not determined			32.47±0.14

Data are expressed as the mean±SD. *P<0.05, significantly different from PBS (no stimulation) within each column; **P<0.05, significantly different from LPS (positive control).
